# Ethylene Participates in the Regulation of Fe Deficiency Responses in Strategy I Plants and in Rice

**DOI:** 10.3389/fpls.2015.01056

**Published:** 2015-11-27

**Authors:** Carlos Lucena, Francisco J. Romera, María J. García, Esteban Alcántara, Rafael Pérez-Vicente

**Affiliations:** ^1^Department of Agronomy, University of CórdobaCórdoba, Spain; ^2^Department of Botany, Ecology and Plant Physiology, University of CórdobaCórdoba, Spain

**Keywords:** ethylene, Fe deficiency responses, iron, regulation, rice, Strategy I, Strategy II

## Abstract

Iron (Fe) is very abundant in most soils but its availability for plants is low, especially in calcareous soils. Plants have been divided into Strategy I and Strategy II species to acquire Fe from soils. Strategy I species apply a reduction-based uptake system which includes all higher plants except the Poaceae. Strategy II species apply a chelation-based uptake system which includes the Poaceae. To cope with Fe deficiency both type of species activate several Fe deficiency responses, mainly in their roots. These responses need to be tightly regulated to avoid Fe toxicity and to conserve energy. Their regulation is not totally understood but some hormones and signaling substances have been implicated. Several years ago it was suggested that ethylene could participate in the regulation of Fe deficiency responses in Strategy I species. In Strategy II species, the role of hormones and signaling substances has been less studied. However, in rice, traditionally considered a Strategy II species but that possesses some characteristics of Strategy I species, it has been recently shown that ethylene can also play a role in the regulation of some of its Fe deficiency responses. Here, we will review and discuss the data supporting a role for ethylene in the regulation of Fe deficiency responses in both Strategy I species and rice. In addition, we will review the data about ethylene and Fe responses related to Strategy II species. We will also discuss the results supporting the action of ethylene through different transduction pathways and its interaction with other signals, such as certain Fe-related repressive signals occurring in the phloem sap. Finally, the possible implication of ethylene in the interactions among Fe deficiency responses and the responses to other nutrient deficiencies in the plant will be addressed.

## Introduction

Iron (Fe) is very abundant in most soils, mainly as Fe^3+^, although its availability to plants is low, especially in high pH calcareous soils (Römheld and Marschner, 1986). On the other hand, excessive Fe accumulation by the plant may lead to toxic effects (Romera et al., 2014; Brumbarova et al., 2015). Therefore, Fe acquisition is highly regulated. Based on the mechanisms developed to facilitate mobilization and uptake of Fe, plants are classified into Strategy I species and Strategy II species. Strategy I species include all higher plants excluding the Poaceae and Strategy II species include the Poaceae (Römheld and Marschner, 1986; Ivanov et al., 2012; Kobayashi and Nishizawa, 2012).

**Figure 1:**
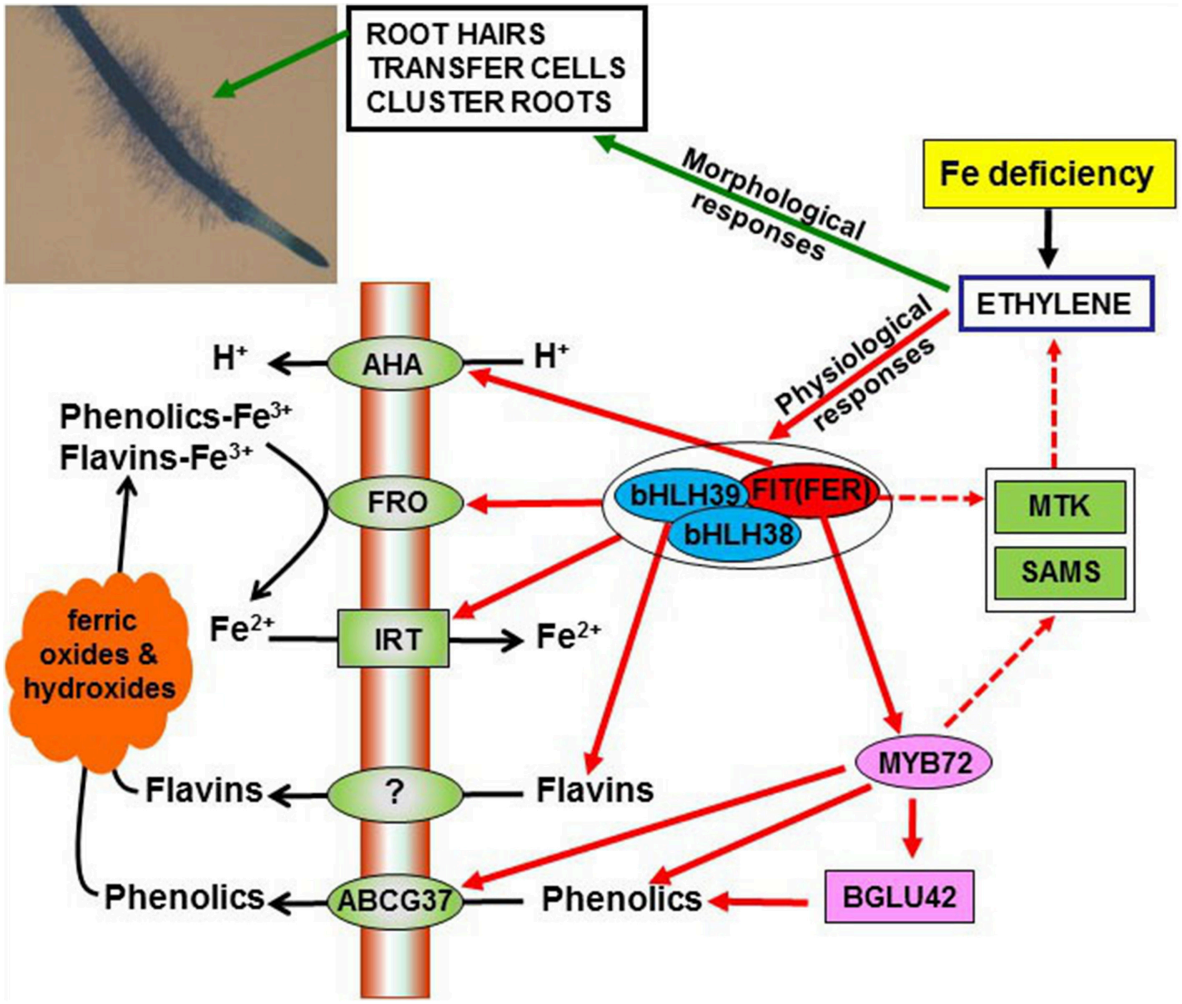
**Overview of the role of ethylene on the regulation of morphological and physiological responses to Fe deficiency in Strategy I species**. Ethylene, through the activation of the transcription factors FIT (FER), bHLH38 and bHLH39, can up-regulate the expression of *FRO* (ferric reductase), *IRT* (iron transporter) and flavin synthesis genes, thus increasing ferric reductase activity, Fe^2+^ uptake and flavin synthesis. Similarly, ethylene, through FIT (FER), can up-regulate *AHA* (H^+^-ATPase) genes, thus causing acidification, and activate the MYB72 transcription factor, which in turn up-regulates genes related to phenolics synthesis. Moreover, MYB72 activates the β-glucosidase BGLU42 and the phenolic efflux transporter ABCG37, both being implicated in the secretion of phenolic compounds. Ethylene has also been implicated in the development of different morphological responses, such as subapical root hairs, root epidermal transfer cells and cluster roots. For the development of these morphological responses, FIT (FER) could indirectly act by affecting ethylene synthesis, through the upregulation of *MTK* and *SAMS* (see **Figure 3**).

The main characteristic of Strategy I species is the necessity for reduction of Fe^3+^ to Fe^2+^, by means of a plasma membrane ferric reductase, prior to its root absorption through a Fe^2+^ transporter (Figure 1; Ivanov et al., 2012; Kobayashi and Nishizawa, 2012). When grown under Fe deficiency, Strategy I species induce several physiological and morphological responses in their roots, that facilitate Fe mobilization to roots and uptake (see Section Role of Ethylene in the Regulation of Fe Deficiency Responses in Strategy I Species).

**Figure 2:**
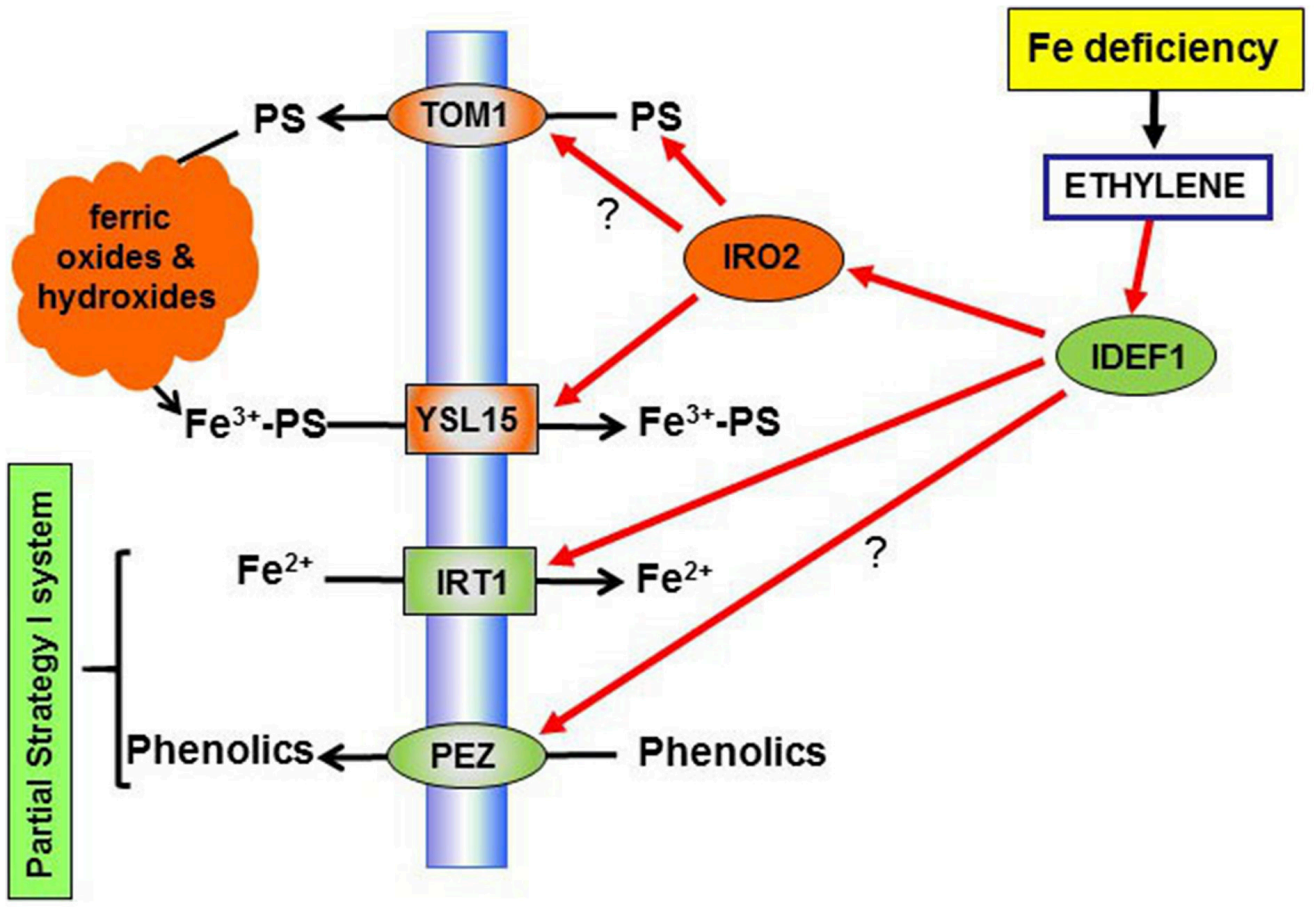
**Overview of the role of ethylene on the regulation of physiological responses to Fe deficiency in rice**. Ethylene, through the subsequent activation of the transcription factors IDEF1 and IRO2, could activate the synthesis of PS (through up-regulation of *NAS* genes; see Figure 3), the expression of the PS efflux transporter TOM1 (not demonstrated yet) and of the PS-Fe^3+^ transporter, YSL15. Moreover, through the activation of the transcription factors IDEF1, ethylene could up-regulate the Fe^2+^ transporter IRT1, and the phenolic efflux transporter PEZ (not demonstrated yet). PS, physotiderophores.

To obtain Fe from the soil, Strategy II species release PS (PhytoSiderophores) from their roots, which form stable Fe^3+^-chelates. These Fe^3+^-chelates (Fe^3+^-PS) are then taken up by specific epidermal root cell plasma membrane transporters (Figure 2; Kobayashi and Nishizawa, 2012). Under Fe-deficient conditions, Strategy II species greatly increase the production and release of PS, the number of Fe^3+^-PS transporters and develop other physiological and regulatory responses (Kobayashi and Nishizawa, 2012; see Section Role of Ethylene in the Regulation of Fe Deficiency Responses in Rice and Strategy II Species). Rice, traditionally considered a Strategy II species (Kobayashi and Nishizawa, 2012), presents some characteristics of Strategy I species, such as enhanced Fe^2+^ uptake through a Fe^2+^ transporter (Figure 2; Ishimaru et al., 2006, 2011; Kobayashi et al., 2014). For this reason, some authors consider it as a plant species that uses a combined strategy (Ricachenevsky and Sperotto, 2014).

Once adequate Fe has been absorbed, Fe deficiency responses need to be down regulated to avoid toxicity and to conserve energy. The regulation of these responses is not fully understood but several hormones and signaling substances have been proposed to participate in their activation, like auxin (Landsberg, 1984), ethylene (Romera and Alcántara, 1994), and NO (nitric oxide; Graziano and Lamattina, 2007), as well as in their suppression, like cytokinins (Séguéla et al., 2008), jasmonic acid (Maurer et al., 2011), and brassinosteroids (Wang et al., 2012). These hypotheses have been mainly focused on Strategy I species while the role of hormones and signaling substances on the regulation of Fe deficiency responses in Strategy II species has been less studied.

In Strategy I species, accumulating evidence supports a role for auxin, ethylene and NO in the activation of Fe deficiency responses through the upregulation of Fe-related genes (Lucena et al., 2006; Graziano and Lamattina, 2007; Waters et al., 2007; Chen et al., 2010; García et al., 2010, 2011; Bacaicoa et al., 2011; Lingam et al., 2011; Meiser et al., 2011; Meng et al., 2012; Wu et al., 2012; Yang et al., 2013, 2014). The implication of all these substances is not unexpected, since auxin, ethylene and NO are closely interrelated (Romera et al., 2011, in press).

This review will focus on the role of ethylene in the regulation of Fe deficiency responses in Strategy I species, although some results related to rice and Strategy II species will also be presented and discussed. We will also review results that suggest interactions among Fe deficiency responses and the responses to other nutrient deficiencies. The possible implication of ethylene in these interactions will also be discussed.

## Ethylene synthesis and signaling under Fe deficiency

**Figure 3:**
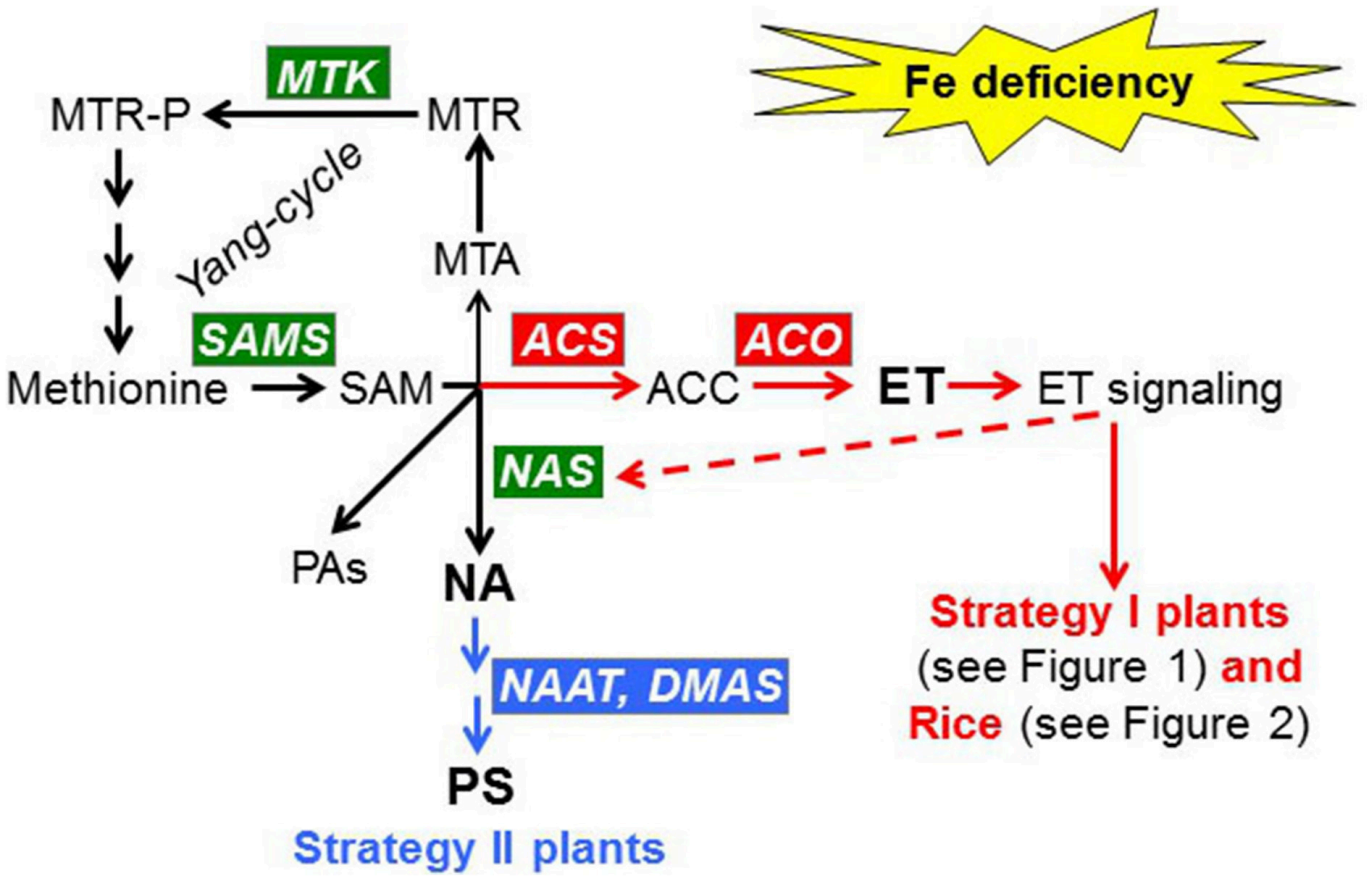
**Effect of Fe deficiency on the synthesis of NA (nicotianamine), PS (phytosiderophores), and ET (ethylene) in Strategy I and Strategy II species**. Fe deficiency induces NA production in both Strategy I and Strategy II species by up-regulating the expression of genes implicated in the Yang-cycle, like *MTK* and *SAMS*, and by up-regulating *NAS* genes (in green). In Strategy I species and Rice, Fe deficiency induces ET production by up-regulating *ACS* and *ACO* genes (in red). ET can then up-regulate *NAS* genes and activate Fe responses in Strategy I plants and Rice. In Strategy II species, Fe deficiency induces PS production by up-regulating the expression of genes implicated in PS synthesis, such as *NAAT* and *DMAS* (in blue). MTK, methylthioribose kinase; SAMS, S-adenosyl metionine synthetase; ACS, ACC synthase; ACO, ACC oxidase; NAS, nicotianamine synthase; NAAT, nicotianamine aminotransferase; DMAS, deoxymugineic acid synthase; PAs, polyamines.

Ethylene is synthesized from methionine via a pathway that requires the enzymes SAMS (SAM synthetases), ACS (ACC synthases) and ACO (ACC oxidades; Figure 3; Sauter et al., 2013). Besides ethylene, SAM (S-adenosyl methionine) is also the precursor for the synthesis of NA (nicotianamine), PAs (polyamines) and PS (phytosiderophores; Figure 3; Kobayashi and Nishizawa, 2012; Sauter et al., 2013). The Yang-cycle allows the recycling of methionine, being MTK (Methyl Thioribose Kinase) one of the enzymes that participates in this cycle (Figure 3; Sauter et al., 2013). Although ethylene's mode of action is not fully understood, a linear signaling pathway has been proposed in Arabidopsis (Shakeel et al., 2013; Wang et al., 2013b):

ET⫣ETreceptors→CTR1⫣EIN2→EIN3/EILs→ERFs→ETresponses

In the absence of ET (ethylene), the kinase CTR1 phosphorylates EIN2 (which is localized to the ER membrane), preventing the cleavage and translocation of the EIN2 C-terminal fragment into the nucleus. In the presence of ethylene, CTR1 is inactivated, resulting in dephosphorylation of EIN2 and its cleavage. The EIN2 C-terminal fragment is then translocated into the nucleus, where it participates in stabilization of the transcription factor EIN3 and downstream gene activation (Shakeel et al., 2013; Wang et al., 2013b). EIN3 belongs to a small family of transcription factors that also includes various EIN3-like proteins: EIL1, EIL2, and EIL3 (Wang et al., 2013b). Mutants of CTR1 present constitutive activation of ethylene signaling, while mutants of EIN2 and EIN3 display reduced sensitivity to ethylene (Shakeel et al., 2013; Wang et al., 2013b). The ERF (Ethylene Response Factor) transcription factors act downstream of EIN3 to activate or repress ethylene-responsive genes although some ERFs can be activated by ethylene-independent transcription factors, not related to EIN3 (Wang et al., 2013b; Thirugnanasambantham et al., 2015).

Fe deficiency can influence both ethylene synthesis and signaling. Additionally, ethylene production can increase upon Fe excess (Yamauchi and Peng, 1995; Li et al., 2015).

### Ethylene synthesis and signaling in strategy I species

Romera et al. (1999) showed that Fe-deficient roots of several Strategy I species produced more ethylene than the Fe-sufficient ones, even before the plants showed any other symptom of deficiency. This excludes that the higher ethylene production could be due to stimulation of wound ethylene in necrotic tissues (Lynch and Brown, 1997). After this report, the higher ethylene production by Fe-deficient roots of different Strategy I species has been confirmed by other authors (Waters and Blevins, 2000; Li and Li, 2004; Molassiotis et al., 2005; Zuchi et al., 2009; Wang et al., 2012; Li et al., 2014a).

The higher ethylene production described for Fe deficiency has been further supported by results showing upregulation of genes implicated in ethylene synthesis, such as *SAMS, ACS*, and *ACO* (Figure 3; García et al., 2010; Stein and Waters, 2012 and references therein; Li et al., 2014a; Moran Lauter et al., 2014; Romera et al., in press and references therein). At the proteomic level, several studies have also shown a significant increase in the SAMS protein (Figure 3) under Fe deficiency (reviewed by López-Millán et al., 2013). Additionally, MTK, an enzyme involved in methionine recycling for a sustained ethylene production (Figure 3), is also induced by Fe deficiency (García et al., 2010; Zamboni et al., 2012; Romera et al., in press and references therein).

Besides ethylene synthesis, Fe deficiency can also affect ethylene responsiveness by altering the expression of genes implicated in ethylene signaling. Several of these genes are upregulated in different Strategy I species under Fe deficiency, like *ETRs* and *ERSs* (coding for ethylene receptors), *EIN2, EIN3, EILs*, and *ERFs* (see above; O'Rourke et al., 2007; García et al., 2010, 2014; Wang et al., 2014a). Whether the expression of these genes enhances or decreases the sensitivity to ethylene is not known yet. It is possible that ethylene sensitivity increases at the earlier stages of Fe deficiency and then decreases as a dampening mechanism, slowing down the ethylene response once it has been initiated. In any case, this deserves further investigation.

### Ethylene synthesis and signaling in rice and strategy II species

There are few publications relating ethylene and Fe deficiency in Strategy II species. Morgan and Hall (1962) showed that Fe-deficient sorghum plants treated with 2,4-D (2,4-dichlorophenoxyacetic acid, a synthetic auxin) produced more ethylene than the Fe-sufficient ones. However, they determined ethylene when plants were very chlorotic and, consequently, the higher ethylene production could be an indirect effect of the advanced stress. After this report, Romera et al. (1999) found that roots from several Fe-deficient Strategy II species (maize, wheat, barley) did not produce more ethylene than the Fe-sufficient ones. Recently, Wu et al. (2011) showed similar results with barley plants. However, Fe-deficient roots from rice, that presents a combined strategy (Figure 2; see Section Introduction), produced more ethylene than the Fe-sufficient ones (Wu et al., 2011). Moreover, several ethylene synthesis genes, like *OsACS, OsACO, OsSAMS*, and *OsMTK*, were up-regulated in rice under Fe deficiency (Figure 3; Kobayashi et al., 2005; Zheng et al., 2009; Kobayashi and Nishizawa, 2012; Itai et al., 2013). Zheng et al. (2009) also found upregulation of a gene coding for a transcription factor relating to ethylene signaling in rice (Os03g64260) under Fe deficiency.

Additionally to rice, *SAMS* and *MTK* genes are also up-regulated in Strategy II species, such as barley and maize, under Fe deficiency (Figure 3; Suzuki et al., 2006; Li et al., 2014b). This is in agreement with the participation of both genes in NA and PS synthesis, besides their participation in ethylene synthesis (Figure 3).

## Role of ethylene in the regulation of Fe deficiency responses in strategy I species

**Figure 4:**
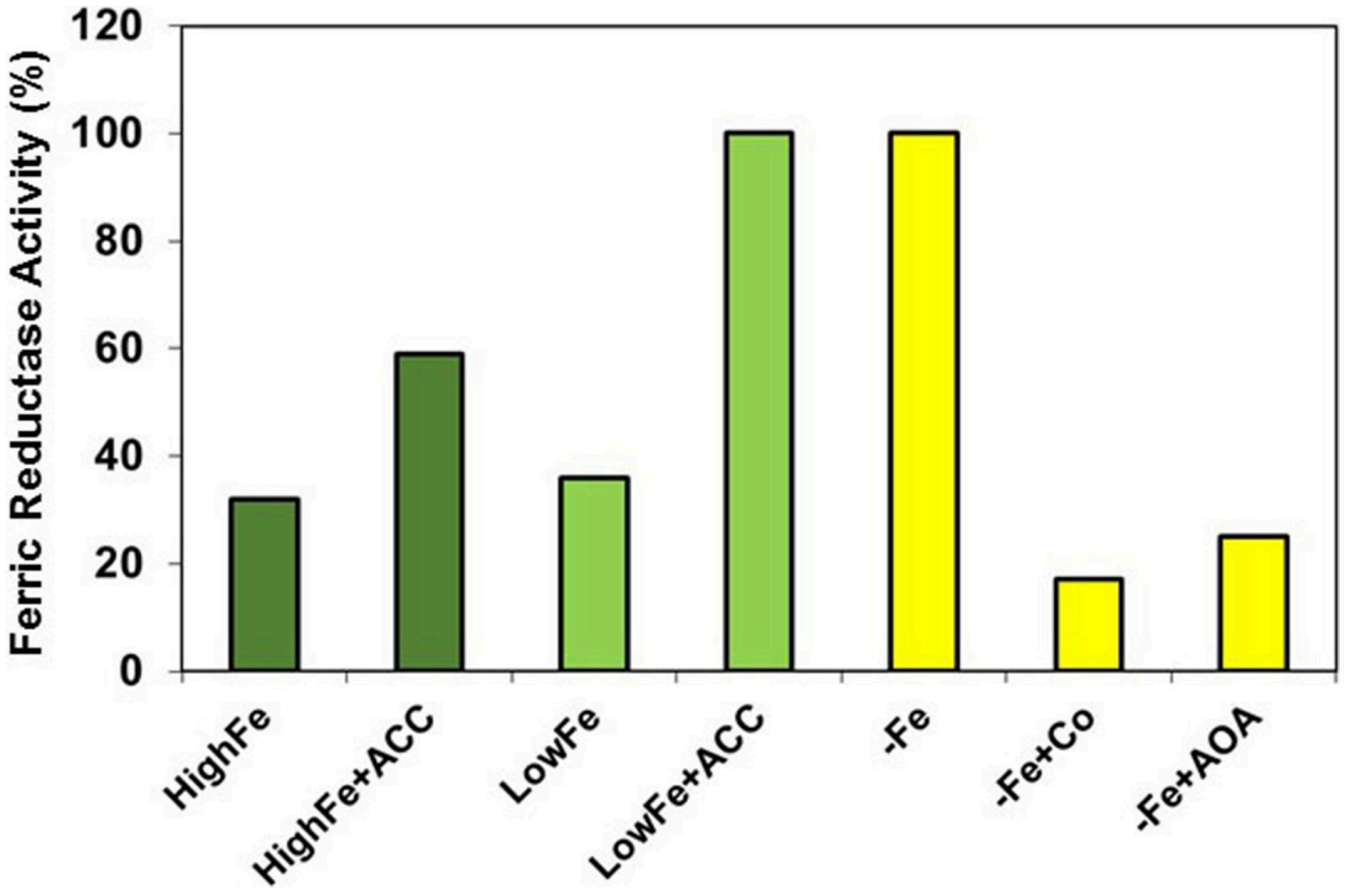
**Effect of ethylene precursors and inhibitors on the ferric reductase activity of Strategy I species**. Ethylene (ACC) activates physiological responses, e.g., ferric reductase activity, to a lesser degree in plants grown with high levels of Fe than in plants grown with low levels. This suggests that ethylene acts in conjunction with some Fe-related repressive signals. On the other hand, ethylene inhibitors, like Co or AOA, block drastically the induction by Fe deficiency of this response, which suggests that ethylene is necessary for its activation. Data of ferric reductase activity expressed as percentage of –Fe plants (re-elaborated from Romera and Alcántara, 1994).

In response to Fe deficiency, Strategy I species induce several physiological and morphological responses in their roots, aimed to facilitate Fe acquisition. These responses are down regulated once Fe uptake is sufficient to meet plants needs, to avoid toxicity and to conserve energy. Romera and Alcántara (1994) showed for the first time that ethylene could be involved in the regulation of both physiological and morphological responses to Fe deficiency in Strategy I species. Both kind of responses work together to effectively increase Fe uptake (Lucena et al., 2006). Consequently, it is not surprising the coordination of their regulation through the participation of the same signal (ethylene) for both of them. Romera and Alcántara (1994), based on the use of ethylene inhibitors and precursors, proposed that Fe deficiency could cause an enhanced production of ethylene and that then ethylene would trigger the activation of both physiological and morphological responses. This hypothesis has been further confirmed by different results, some of them already considered in previous reviews (Romera and Alcántara, 2004; Romera et al., 2007). In addition to the higher ethylene production of Fe-deficient roots (see previous Section), other results also support a role for ethylene in the regulation of Fe deficiency responses in Strategy I species. These other results are based on a variety of experimental approaches, such as the use of ethylene inhibitors, like Co (cobalt), AOA (aminooxyacetic acid), AVG (aminoethoxyvinylglycine) or STS (silver thiosulfate), the ethylene precursor ACC (1-aminocyclopropane-1-carboxylic acid), the ethylene-releasing substance ethephon, ethylene itself, ethylene mutants (ethylene insensitive, ethylene constitutive or ethylene overproducers), and molecular biology techniques, such as transgenic lines, transcriptomics, proteomics, bimolecular fluorescence complementation, and yeast two-hybrid (Romera and Alcántara, 1994, 2004; Schmidt et al., 2000b; Schmidt and Schikora, 2001; García et al., 2010; Lingam et al., 2011; Meiser et al., 2011; López-Millán et al., 2013; Yang et al., 2014). Ethylene, whose production increases under Fe deficiency, acts as activator of most Fe deficiency responses. Consequently, ethylene inhibitors block the responses while ethylene itself or ethylene precursors (ACC or ethephon) promote them (Figure 4; Romera and Alcántara, 1994, 2004; Molassiotis et al., 2005; Lingam et al., 2011).

Here, we will review the more recent results supporting a role for ethylene in the regulation of both physiological and morphological responses to Fe deficiency in Strategy I species, most of them obtained with molecular biology techniques.

### Role of ethylene on physiological responses

As previously stated, Strategy I species need to reduce Fe^3+^, the most abundant form in soils, to Fe^2+^, prior to uptake. This reduction is mediated by a plasma membrane ferric reductase (encoded by *AtFRO2* in Arabidopsis; Robinson et al., 1999) and then Fe^2+^ is taken up by a Fe^2+^ transporter (encoded by *AtIRT1* in Arabidopsis; Eide et al., 1996). Ferric reductases and iron transporter genes have also been cloned from other plant species, like tomato (Eckhardt et al., 2001; Li et al., 2004), pea (Waters et al., 2002; Cohen et al., 2004), and cucumber (Waters et al., 2007). When grown under Fe deficiency, Strategy I species enhance both ferric reductase activity (due to increased expression of *AtFRO2*-like genes) and Fe^2+^ uptake capacity (due to increased expression of *AtIRT1*-like genes) (Walker and Connolly, 2008; Kobayashi and Nishizawa, 2012).

In addition to enhanced ferric reductase activity and Fe^2+^ uptake capacity, Strategy I species can develop other Fe deficiency responses aimed to facilitate Fe mobilization and uptake from the soil. This includes the capacity to acidify the rhizosphere medium (due to increased expression of plasma membrane proton-ATPase genes, such as *AtAHA2, AtAHA7, CsHA1*, and *MxHA7*), which contributes to Fe solubilisation (Santi et al., 2005; Waters et al., 2007; Zha et al., 2014); and the increased synthesis and release of Fe^3+^-related compounds such as flavins and phenolics (Jin et al., 2007; Rodríguez-Celma et al., 2013; Fourcroy et al., 2014; Schmid et al., 2014; Schmidt et al., 2014). The exact function of flavins and phenolics is not totally clear, but it has been proposed that they could act as “Iron Binding Compounds” contributing to Fe mobilization in the rhizosphere (Rodríguez-Celma and Schmidt, 2013). Alternatively, their main function could be related to Fe^3+^reduction, acting as long distance electron shuttle (Rodríguez-Celma and Schmidt, 2013).

**Figure 5:**
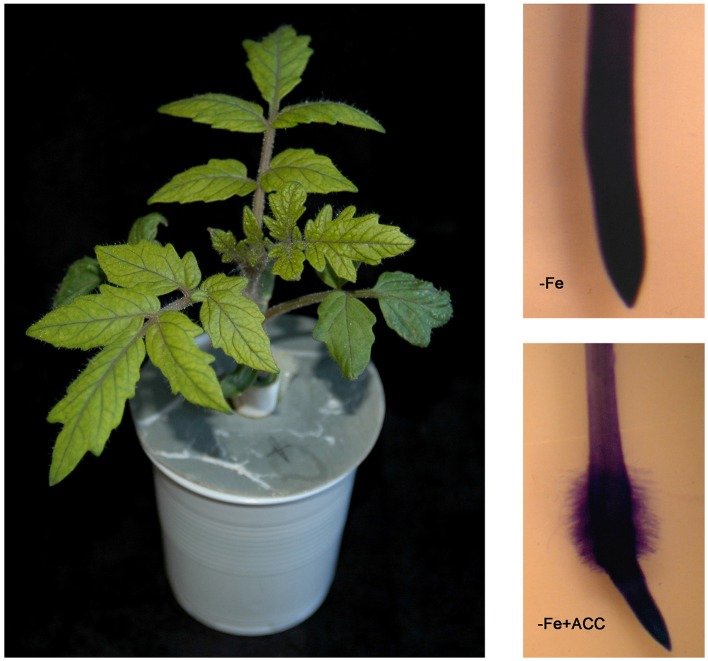
**Subapical root hairs in the tomato *fer* mutant**. The tomato *fer* mutant (left) does not develop either subapical root hairs (right, top; Romera and Alcántara, 2004) or root transfer cells (Schmidt et al., 2000a) when grown under Fe deficiency. However, it develops both root hairs (right, bottom; Romera and Alcántara, 2004; see Supplementary Material) and transfer cells (Schmidt et al., 2000a) upon ACC treatment. This suggests that FER could influence these morphological responses through its possible indirect effect on ethylene synthesis (see Figure 1).

The regulation of the Fe-related genes described above is not totally understood but in the last years several transcription factors that participate in their activation have been found. The master regulator of most of the responses to Fe deficiency is the tomato SlFER, identified as a bHLH transcription factor (Ling et al., 2002), or its homologs AtFIT in *Arabidopsis* (bHLH29; Colangelo and Guerinot, 2004; Jakoby et al., 2004; Bauer et al., 2007) and MxFIT in *Malus xiaojinensis* (Yin et al., 2014). The tomato *fer* mutant (Figure 5) and the Arabidopsis *fit* mutant are very chlorotic and lack the ability to activate most Fe responses in roots (Brown et al., 1971; Ling et al., 2002; Colangelo and Guerinot, 2004; Jakoby et al., 2004). In Arabidopsis, the AtFIT regulatory network comprises other bHLH transcription factors, such as AtbHLH38, AtbHLH39, AtbHLH100, and AtbHLH101. All of them have redundant functions and can interact with AtFIT to form heterodimers that activate the expression of the Fe acquisition genes *AtFRO2* and *AtIRT1* (Figure 1; Yuan et al., 2008; Wang et al., 2013a; Maurer et al., 2014; Brumbarova et al., 2015). FIT(FER) is induced in roots in response to Fe deficiency while the other bHLHs are induced in both roots and leaves in response to Fe deficiency (Colangelo and Guerinot, 2004; Jakoby et al., 2004; Brumbarova and Bauer, 2005; Wang et al., 2007; Yuan et al., 2008; Brumbarova et al., 2015). In addition to the Arabidopsis AtFIT regulatory network, the AtPYE (AtPOPEYE) regulatory network has been described and implied in the regulation of a different subset of stele expressed Fe-related genes (Long et al., 2010; Ivanov et al., 2012; Brumbarova et al., 2015; Zhang et al., 2015). Other transcription factors related to Fe deficiency responses in Arabidopsis are AtMYB72 and AtMYB10 (Colangelo and Guerinot, 2004; Palmer et al., 2013; Zamioudis et al., 2014). They have redundant functions and have been implicated in the Fe deficiency induced up-regulation of *AtNAS4* (encoding NicotianAmine Synthase), playing an opposite role to AtPYE (Palmer et al., 2013).

**Figure 6:**
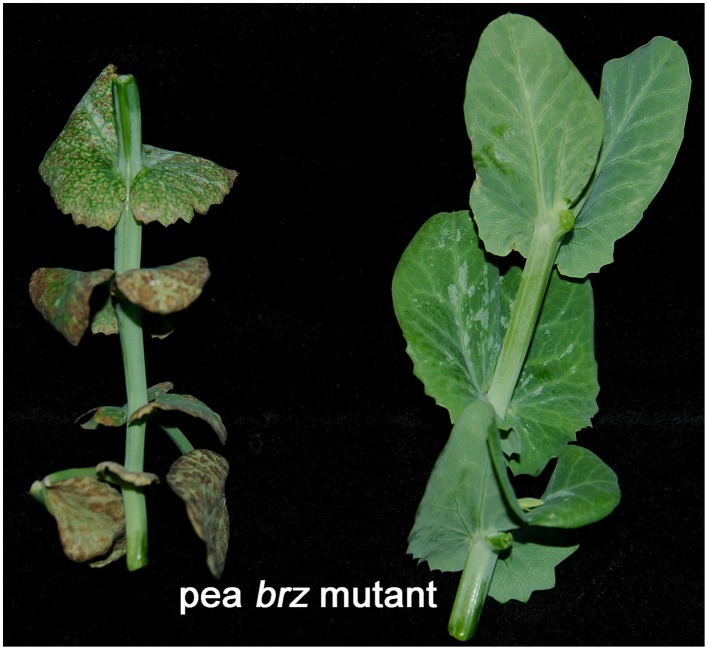
**The pea *brz* mutant can accumulate toxic levels of Fe in its leaves**. Some mutants that present constitutive activation of Fe physiological responses, like the pea *brz* mutant (above; Romera et al., 1996) and the pea *dgl* mutant (Romera et al., 2014), can accumulate high levels of Fe and other metals in their leaves (Romera et al., 1996, 2014), which causes toxicity symptoms (left). Upon treatment with ethylene inhibitors, the Fe physiological responses are blocked and the concentration of Fe accumulated in the leaves diminish (Romera et al., 1996, 2014; see Supplementary Material). Consequently, the toxicity symptoms disappear (right). The *brz* plants (25-d-old) were transferred to nutrient solution with 20 μM FeEDDHA (left) or to nutrient solution with 20 μM FeEDDHA and the ethylene inhibitor AOA (at 20 μM; right) during 7 days.

The addition of ethylene inhibitors to Fe-deficient plants inhibits the induction of most of their physiological Fe responses, such as ferric reductase activity (Figure 4), Fe^2+^ uptake capacity, rhizosphere acidification, and flavin excretion (Romera and Alcántara, 1994, 2004; Lucena et al., 2006; Romera et al., 2007; Waters et al., 2007). The ferric reductase activity and other Fe responses are also drastically inhibited by ethylene inhibitors in mutants that show constitutive Fe responses and, as a consequence, accumulate toxic concentrations of Fe in their leaves, like the pea *brz* (*bronze*) and *dgl* mutants. Upon treatment with ethylene inhibitors, these mutants lower Fe uptake and, consequently, avoid Fe toxicity symptoms (Figure 6; Romera et al., 1996, 2014; see Supplementary Material). In contrast to ethylene inhibitors, the addition of ethylene, ACC or ethephon to Fe-sufficient plants induces some physiological Fe responses, such as enhanced ferric reductase activity (Figure 4), located in the subapical regions of the roots where formation of root hairs is also induced (Figure 5; Romera and Alcántara, 2004; Lucena et al., 2006; Waters et al., 2007; Romera et al., 2007; García et al., 2010, 2011). In supporting the role of ethylene, it should be mentioned that Fe-efficient cultivars of pea (Kabir et al., 2012) and *Medicago truncatula* (Li et al., 2014a) produce more ethylene than the Fe-inefficient ones. This suggests that the higher ethylene production would allow these cultivars to better activate their responses to Fe deficiency.

**Figure 7:**
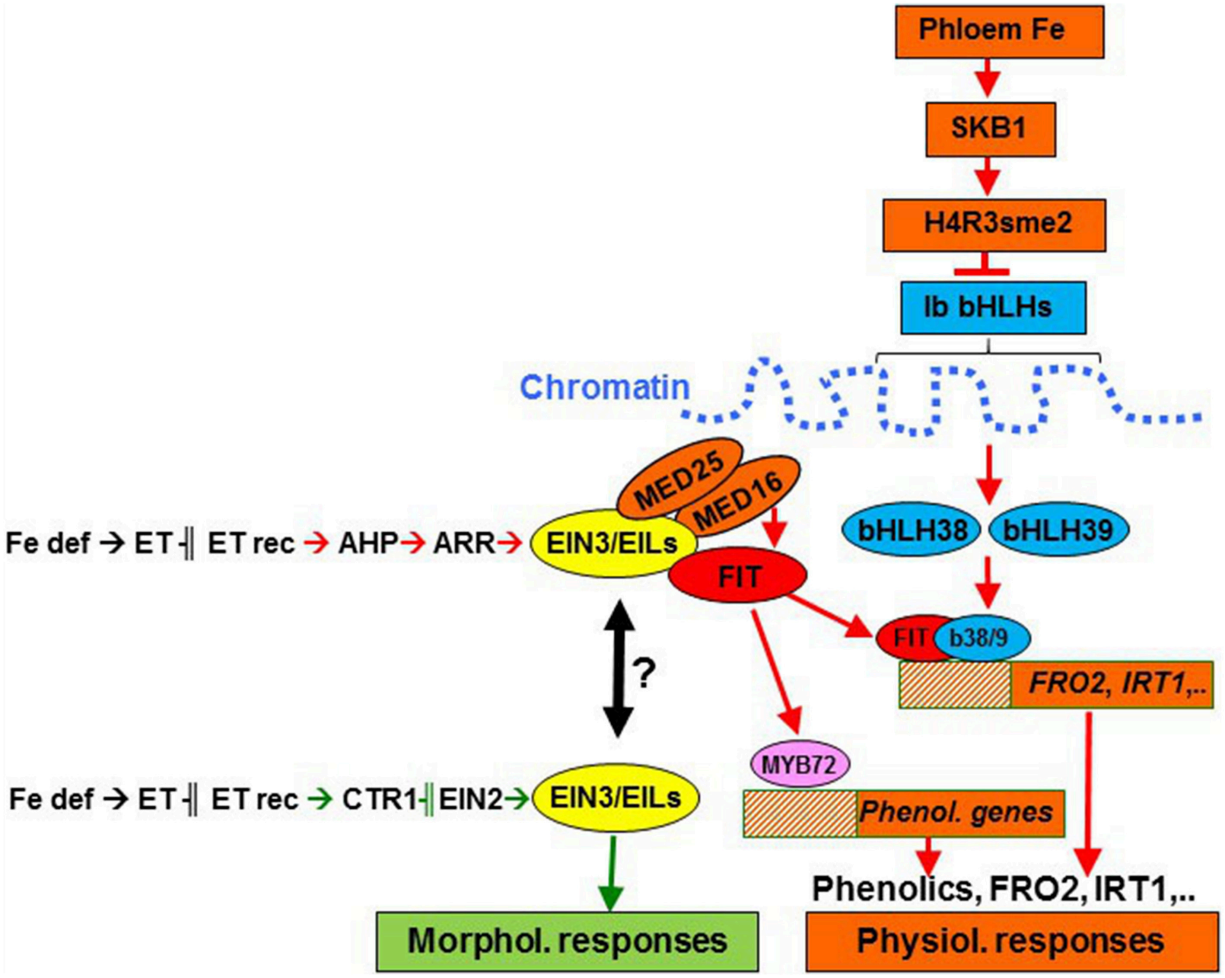
**Ethylene may regulate morphological and physiological responses to Fe deficiency through different signaling pathways in Strategy I species**. Ethylene could regulate morphological responses through a pathway including CTR1 and EIN2 (in green) and physiological responses through a CTR1-EIN2-independent pathway (in red), instead using AHPs (phosphotransfer proteins) and ARRs (response regulators; Shakeel et al., 2013). Both pathways could converge through EIN3/EILs activity under certain circumstances, since EIN3 and EIL transciption factors have been involved in the regulation of both physiological (Lingam et al., 2011; Yang et al., 2014) and morphological responses, e.g., root hairs (Zhu et al., 2011) and transfer cells (Andriunas et al., 2011). For the regulation of physiological responses, ethylene may interact with some Fe-related repressive signals, probably moving through the phloem (García et al., 2013; Mendoza-Cózatl et al., 2014; Zhai et al., 2014). This phloem-Fe, through SKB1, could favor the chromatin package, where are located the bHLH38 and bHLH39 genes, thus inhibiting their transcription (Fan et al., 2014). Fe def, Fe deficiency; ET rec, ethylene receptors.

The participation of ethylene in the activation of physiological responses is further supported when analyzing its effects on the genes controlling these responses (Lucena et al., 2006; Waters et al., 2007; García et al., 2010; Lingam et al., 2011; Yang et al., 2014; Romera et al., in press). Ethylene up-regulates the expression of the key transcription factors AtFIT (or its tomato homolog SlFER), AtbHLH38, AtbHLH39, and AtMYB72 (Figure 1; Lucena et al., 2006; García et al., 2010, 2013, 2014; Lingam et al., 2011). The link between ethylene and AtFIT has been recently reinforced. It has been shown that AtEIN3 and AtEIL1, two transcription factors in the ethylene signaling pathway (see Section Ethylene Synthesis and Signaling under Fe Deficiency), interact with AtMED16 (Mediator) and AtMED25, to form a complex implicated in the transcription of *AtFIT* (Figure 7; Yang et al., 2014; Zhang et al., 2014; Brumbarova et al., 2015). Moreover, AtEIN3 and AtEIL1 can also influence the posttranscriptional stability of AtFIT (Lingam et al., 2011). AtbHLH38 and AtbHLH39 interact with AtFIT to form heterodimers that activate the expression of the Fe acquisition genes *AtFRO2* and *AtIRT1* (Figures 1, 7; Yuan et al., 2008; Wang et al., 2013a). Consequently, the ferric reductase activity (depending on *AtFRO2*-like genes) and the Fe^2+^ uptake capacity (depending on *AtIRT1*-like genes) can be regulated by ethylene through AtFIT, AtbHLH38, and AtbHLH39. Similarly, the acidification capacity (depending on *AtAHA*-like genes, also activated by AtFIT-like transcription factors; Colangelo and Guerinot, 2004) can be regulated by ethylene too (Figure 1; Romera and Alcántara, 1994, 2004; Waters et al., 2007). Flavin excretion is another response inhibited by ethylene inhibitors (Romera and Alcántara, 2004). Since AtbHLH38 and AtbHLH39 have been implicated in the activation of flavin production (Vorwieger et al., 2007), and both transcription factors can be induced by ethylene (García et al., 2010), these results reinforce the hypothesis that flavin production is also controlled by ethylene (Figure 1). In relation to the excretion of phenolics, another response to Fe deficiency (Römheld and Marschner, 1986), some indirect studies suggest that the biosynthesis of some phenolics, like caffeic, coumaric and ferulic acids, could be stimulated by ethylene through an increase in the activity of the phenylalanine ammonia lyase enzyme (Rhodes and Wooltorton, 1973; Heredia and Cisneros-Zevallos, 2009; Liang et al., 2013). Very recently, Zamioudis et al. (2014) have found that the β-glucosidase AtBGLU42 is very important for the secretion of phenolic compounds under Fe deficiency. Furthermore, they have shown that the expression of *AtBGLU42*, as well as the expression of the phenolic efflux transporter *AtABCG37* (formerly named *AtPDR9*) and other genes involved in phenolic synthesis, are dependent on the transcription factor AtMYB72 (Figure 1; Zamioudis et al., 2014). Since *AtMYB72* expression is activated by ethylene (García et al., 2010), probably through AtFIT (AtMYB72 is one of its targets; Sivitz et al., 2012), this suggests that ethylene can also regulate phenolic secretion (Figure 1). In supporting this view, it should be noted that *AtBGLU42* expression is also activated by ethylene (García et al., 2010).

Besides genes related to Fe acquisition from the growth medium, there are other genes that are induced under Fe deficiency in roots and/or leaves, like *AtFRD3* (Ferric Reductase Defective3; Rogers and Guerinot, 2002) and *AtNASs* (NicotianAmine Synthase; Klatte et al., 2009), that are very important for internal Fe mobilization and homeostasis. The FRD3 protein belongs to the multidrug and toxin efflux (MATE) family and has been implicated in the loading of citrate into the xylem, which is necessary for Fe translocation from roots to shoots (Rogers and Guerinot, 2002; Durrett et al., 2007; Roschzttardtz et al., 2011). The higher expression of *FRD3* under Fe deficiency is generally associated with increased synthesis of organic acids, like citrate and malate (Landsberg, 1981; Kabir et al., 2012; Li et al., 2014a). *In toto*, both responses cooperate for the internal translocation of Fe from roots to shoots. NAS (NA Synthase) proteins participate in the synthesis of NA (nicotianamine), which is a chelating agent implied in the long-distance transport of Fe (and other metals) and that facilitates the transport of Fe through the phloem to sink organs (Klatte et al., 2009; Schuler et al., 2012). NA is also the precursor for the biosynthesis of PS, that play a key role in Strategy II species (Figure 3; Kobayashi and Nishizawa, 2012). The mutants related to these genes (Arabidopsis *frd3* and *nas4x*, and the tomato *NAS* mutant *chloronerva*) show constitutive activation of Fe responses, even when grown under Fe-sufficient conditions (Rogers and Guerinot, 2002; Klatte et al., 2009; García et al., 2013). This suggests that the precise distribution of Fe throughout the plant has a decisive role in the control of Fe deficiency responses. Ethylene has been implicated in the activation of both *AtNAS* (*AtNAS1* and *AtNAS2*) and *AtFRD3* genes (García et al., 2010).

### Role of ethylene on morphological responses

In addition to physiological responses, Strategy I species can develop some morphological responses in their roots under Fe deficiency, like subapical root hairs (Figure 1), root epidermal transfer cells and cluster roots (also named proteoid roots; Kramer et al., 1980; Römheld and Marschner, 1986; Romera and Alcántara, 1994, 2004; Schmidt et al., 2000a; Waters and Blevins, 2000; Schmidt and Schikora, 2001; Schikora and Schmidt, 2002; Zaid et al., 2003; Romera et al., 2007; García et al., 2015). Transfer cells have increased surface area, due to invaginations of the plasma membrane (Kramer et al., 1980; Schmidt et al., 2000a; Schikora and Schmidt, 2002). Proteoid roots are clusters of closely spaced short lateral rootlets formed in some plant species adapted to poor soils (Waters and Blevins, 2000; Zaid et al., 2003; Wang et al., 2014b). Most of these root modifications are also formed under P deficiency since both Fe and P may be poorly available in soils (Schmidt and Schikora, 2001; Schikora and Schmidt, 2002; Wang et al., 2014b). Root hairs, root epidermal transfer cells and cluster roots enhance nutrient uptake by increasing the surface of contact of roots with soil and by chemically modifying the soil environment (Wang et al., 2014b). Besides these specialized morphological responses, Fe deficiency, depending on the extent of the deficiency (mild, moderate, or severe), can also change root system architecture by altering the number, length, and diameter of roots. Generally, Fe deficient plants exhibit a shallower architecture that results from inhibition of primary root elongation (Kramer et al., 1980). Additionally, Fe deficiency can cause an increase in lateral root density (Kramer et al., 1980; Jin et al., 2008).

Ethylene and auxin, along with other hormones and signaling substances, have been implicated in all the morphological changes described in the above paragraph (Romera and Alcántara, 1994, 2004; Schmidt et al., 2000a; Waters and Blevins, 2000; Schmidt and Schikora, 2001; Schikora and Schmidt, 2002; Zaid et al., 2003; Romera et al., 2007; Muday et al., 2012; Wang et al., 2014b; García et al., 2015). Both hormones synergistically inhibit root elongation while play an antagonistic role on lateral root formation (Muday et al., 2012). Moreover, ethylene influences auxin distribution at the root tip, thus affecting the development of subapical root hairs (Muday et al., 2012; Lee and Cho, 2013).

The implication of ethylene on the regulation of morphological responses to Fe deficiency has been based on the use of ethylene inhibitors and precursors, and the use of ethylene mutants. The addition of ethylene inhibitors to Fe-deficient plants inhibited subapical root hairs, transfer cells and cluster roots, while the addition of ethylene or ethylene precursors (ACC or ethephon) to Fe-sufficient plants promoted them (Romera and Alcántara, 1994, 2004; Schmidt et al., 2000a; Schmidt and Schikora, 2001; Schikora and Schmidt, 2002; Zaid et al., 2003; Romera et al., 2007). In the same way, some ethylene insensitive mutants, like the *Arabidopsis etr1* and *ein2*, the soybean *etr1* and the *Medicago truncatula sickle*, did not develop subapical root hairs either under Fe-deficiency or upon ACC treatment, while the wild-types did (reviewed in Romera and Alcántara, 2004). By contrast, the *Arabidopsis* ethylene constitutive mutant *ctr1* developed subapical root hairs even under Fe-sufficient conditions (Romera and Alcántara, 2004).

The implication of ethylene on the development of transfer cells has also been reinforced by some studies with cotyledons. Ethylene functions as a key inductive signal for wall ingrowth and, consequently, transfer cell formation in epidermal cells of cotyledons (Zhou et al., 2010) while glucose functions as a negative regulator (Andriunas et al., 2011). Glucose modulates the amplitude of the ethylene-stimulated wall ingrowth induction by down-regulating the expression of ethylene synthesis and signaling genes, such as *EIN3/EILs* (Andriunas et al., 2011). These antagonistic effects of ethylene and glucose suggest that EIN3/EILs (see Section Ethylene Synthesis and Signaling under Fe Deficiency) act as integrators of glucose and ethylene regulation of transfer cell formation.

### Are physiological and morphological responses regulated similarly by ethylene?

Since ethylene has been implicated in the regulation of most of the physiological and morphological responses to Fe deficiency in Strategy I species (Figures 1, 7), a question emerged: Are all of them regulated by ethylene in the same way? The answer to this question, based on results with ethylene mutants, is that different responses can be regulated by ethylene through different signaling pathways. On the other hand, many results suggest that ethylene acts in conjunction with other hormones and signaling substances to regulate the responses. Some of these signals (auxin, nitric oxide) act positively on the regulation of Fe deficiency responses in Strategy I species while other ones (ABA, cytokinins, jasmonic acid, brassinosteroids) act negatively (Giehl et al., 2009; Hindt and Guerinot, 2012; Brumbarova et al., 2015; García et al., 2015; Romera et al., in press).

**Figure 8:**
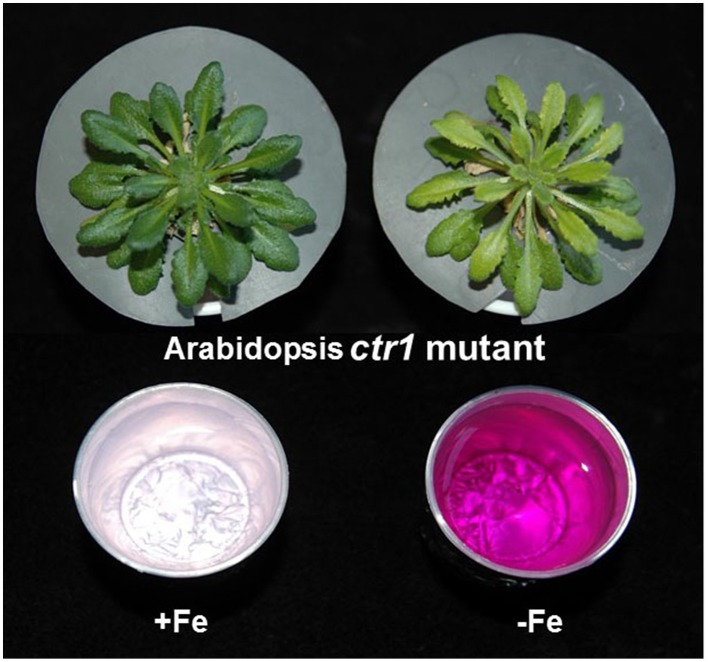
**Induction of ferric reductase activity in the Arabidopsis ethylene constitutive mutant *ctr1* under Fe deficiency**. Neither the Arabidopsis ethylene constitutive mutant *ctr1* (above; García et al., 2014) nor the Arabidopsis ethylene overproducer mutant *eto* (Schmidt et al., 2000b) present constitutive activation of physiological responses, like ferric reductase activity, when grown under Fe-sufficient conditions (Schmidt et al., 2000b; García et al., 2014; see Supplementary Material). This suggests that ethylene can not activate these responses until some Fe-related repressive signals are eliminated (see Figures 4, 7). Some *ctr1* plants (45-d-old) grown in nutrient solution with Fe were transferred to nutrient solution without Fe during the last 7 days. After that, ferricreductase activity was determined (García et al., 2014). The ferric reductase activity is enhanced under Fe deficiency and denoted by the purple color of the assay solution (right, bottom).

Results from ethylene insensitive mutants suggest that physiological and morphological responses can be regulated through different signaling pathways. For instance, the development of subapical root hairs is impaired in the Arabidopsis ethylene insensitive mutants *ein2* and *etr1*, either under Fe deficiency or upon ACC treatment (Schmidt and Schikora, 2001; Romera and Alcántara, 2004), while the enhanced ferric reductase activity and the expression of Fe acquisition genes is not impaired (Lucena et al., 2006; García et al., 2010). On the other hand, both the Arabidopsis ethylene constitutive mutant *ctr1* and the Arabidopsis ethylene overproducer mutant *eto* have constitutive subapical root hairs (a Fe deficiency response) in complete nutrient solution; however, neither of these mutants have full constitutive activation of Fe physiological responses (Figure 8; Schmidt et al., 2000b; Romera and Alcántara, 2004; García et al., 2007, 2014, 2015; see Supplementary Material). In the same way, root hairs, transfer cells and cluster roots are almost fully induced by ACC or ethephon in plants grown with high levels of Fe while physiological responses are activated to a lesser degree than when applied to plants grown with low levels, or in absence, of Fe (Figure 4; Romera and Alcántara, 1994; Schmidt et al., 2000a; Zaid et al., 2003; Lucena et al., 2006; García et al., 2013).

From the results above, several conclusions can be drawn. First, morphological and physiological responses can be differently regulated by ethylene. Second, for some morphological responses, like root hairs, ethylene acts through a signaling pathway including ETR1, CTR1, and EIN2 (Figure 7). Third, for some physiological responses, like ferric reductase activity, ethylene could act through a pathway where EIN2, and possibly CTR1, are not strictly required (Figure 7). Fourth, for the regulation of physiological responses, ethylene could act in conjunction with Fe-related repressive signals since the *ctr1* and *eto* mutants do not have full constitutive activation of these responses (see above).

The existence of an alternate route for ethylene signaling, besides the conventional one including CTR1 and EIN2 (Figure 7; Shakeel et al., 2013), is further supported by results showing that the Arabidopsis *ctr1* and *ein2* mutants respond to both ACC (García et al., 2010, 2014) and ethylene inhibitors (García et al., 2007) for physiological responses. Does this mean that CTR1 and EIN2 do not participate at all in the regulation of these responses? Some experimental results suggest that, although not strictly required, CTR1 and EIN2 can participate in this regulation. As examples, hypoxia inhibited physiological responses in Fe-deficient wild type plants but not in *ctr1* mutant plants (García et al., 2014), which suggests that CTR1 can play a role in their regulation. A possible explanation for the participation of CTR1 and EIN2, without being strictly required, could be the possible interaction between the conventional route for ethylene signaling and the alternate one under certain circumstances. In such cases, both routes could converge downstream through EIN3/EILs (Figure 7). In supporting this view, it should be noted that EIN3/EILs transcription factors have been involved in the up-regulation of FIT (activator of most physiological responses; Figure 7; Yang et al., 2014; Brumbarova et al., 2015) but also in the development of morphological responses, such as root hairs (Figure 7; Zhu et al., 2011) and transfer cells (Andriunas et al., 2011).

In addition to physiological responses (Figures 1, 7), FER (FIT homolog) has also been implicated in the activation of morphological responses. The tomato *fer* mutant does not develop either subapical root hairs or transfer cells under Fe deficiency (Schmidt et al., 2000a). However, it does develop both subapical root hairs (Figure 5; Romera and Alcántara, 2004; see Supplementary Material) and root transfer cells (Schmidt et al., 2000a) upon ACC treatment. This suggests that FER could indirectly activate morphological responses by influencing ethylene synthesis. This suggestion is further supported by two experimental results. First, the *AtMTK* gene, implicated in ethylene synthesis (Figure 3), is activated by ethylene through *AtFIT* (Figure 1; Colangelo and Guerinot, 2004; García et al., 2010). Second, one *AtSAMS* gene implicated in ethylene synthesis (Figure 3) is up-regulated by the AtMYB72 transcription factor (Zamioudis et al., 2014), that is a direct target of AtFIT (Figure 1; Sivitz et al., 2012).

In relation to the existence of Fe-related repressive signals, we can speculate that since physiological responses are not fully activated by ACC in “high Fe” plants (Figure 4), and are not fully activated in Fe-sufficient *ctr1* (Figure 8) and *eto* mutants, some Fe-related signals are acting negatively to block physiological responses. It has been proposed that these Fe-related repressive signals are not associated with the total Fe in roots but with some Fe compound(s) moving through the phloem, which could negatively interact with ethylene signaling to regulate Fe physiological responses (García et al., 2013; Mendoza-Cózatl et al., 2014; Zhai et al., 2014; Romera et al., in press). This does not preclude that these signals can also affect ethylene synthesis.

A possible step affected by the Fe-related repressive signals would be the Shk1 binding protein 1 (SKB1/AtPRMT5) in Arabidopsis (Fan et al., 2014). SKB1 associates with the chromatin of the Ib subgroup bHLH genes (AtbHLH38, AtbHLH39, AtbHLH100, and AtbHLH101) and symmetrically dimethylates histone H4R3, thereby increasing H4R3sme2 and inhibiting transcription (Figure 7). The quantity of SKB1 that associates with the chromatin of these bHLH genes depends on the Fe status of the plants, in such a way that Fe sufficiency increases its association, thus inactivating the transcription of the Ib subgroup bHLH genes (Figure 7; Fan et al., 2014; Brumbarova et al., 2015). The possible effect of the Fe-related repressive signals through SKB1 would explain several experimental results. First, it would explain why the overexpression of *AtFIT* does not activate physiological responses in plants grown with high levels of Fe (Colangelo and Guerinot, 2004; Jakoby et al., 2004) while the overexpression of *AtFIT* with either *AtbHLH38* or *AtbHLH39* does (Yuan et al., 2008). Second, it would explain why the foliar application of Fe inhibits more drastically the expression of *AtbHLH38* and *AtbHLH39* than that of *AtFIT* (García et al., 2013). Third, it would explain why Fe physiological responses are not fully activated in Fe-sufficient *ctr1* (Figure 8) and *eto* mutants. Finally, it would explain why the application of ACC to Fe-sufficient plants does not fully activate physiological responses (Figure 4; Romera and Alcántara, 1994; Lucena et al., 2006; García et al., 2013). Taken together, the results described above indicate that ethylene can not activate Fe physiological responses until some Fe-related repressive signal is removed. This combinatorial control would provide Fe-specificity to the system, suggesting that both Fe deficiency and ethylene action are necessary for full transcriptional activation (Lucena et al., 2006).

## Role of ethylene in the regulation of Fe deficiency responses in rice and strategy II species

To our knowledge, specialized morphological responses to Fe deficiency have not been described in Strategy II species, although they can change their root system architecture. As example, Fe-deficient maize plants developed more lateral roots than Fe-sufficient ones (Li et al., 2014b). Therefore, here we will only describe the role of ethylene in the regulation of physiological responses to Fe deficiency in Strategy II species.

The Strategy II response relies on biosynthesis and secretion of PS (PhytoSiderophores), of the MA (Mugineic Acid) family, which are synthesized from SAM (*S*-Adenosyl Methionine; Figure 3). This pathway includes three sequential enzymatic reactions mediated by NAS (NA Synthase), NAAT (NA AminoTransferase), and DMAS (DeoxyMugineic Acid Synthase; Figure 3; Kobayashi and Nishizawa, 2012). Some years ago, Nozoye et al. (2011) identified the PS efflux transporter OsTOM1 (Transporter Of MAs 1) from rice and the PS efflux transporter HvTOM1 from barley. The Fe^3+^-PS are taken up into root cells by Fe^3+^-PS transporters, like ZmYS1 (YELLOW STRIPE 1) and YSL (YELLOW STRIPE 1–like) transporters (Figure 2; Curie et al., 2001; Inoue et al., 2009).

Rice, despite being traditionally considered a Strategy II species (Kobayashi and Nishizawa, 2012), possesses some characteristics of Strategy I species, such as a Fe^2+^ transporter, OsIRT1, which allows it to absorb Fe^2+^ from the soil, in addition to its Strategy II-based Fe^3+^-PS uptake system, and its capacity to release phenolics (Figure 2; Ishimaru et al., 2006). Some authors consider rice as a plant species that uses a combined strategy (Ricachenevsky and Sperotto, 2014; see Section Introduction).

Under Fe deficiency, Strategy II species induce the expression of genes implicated in PS synthesis (*NAS, NAAT*, and *DMAS*) as well as genes implicated in PS efflux (*TOM1*) and Fe^3+^-PS uptake (YS1, YSL15; Figures 2, 3; Kobayashi and Nishizawa, 2012; Itai et al., 2013; Kobayashi et al., 2014). Additionally, Strategy II species, similarly to Strategy I species, increase the synthesis of organic acids under Fe deficiency (Landsberg, 1981). Rice also induces the expression of the *OsIRT1* gene (Ishimaru et al., 2006) and increases the production and release of phenolic compounds to the rhizosphere, through the OsPEZ1 phenolic efflux transporter (Figure 2; Ishimaru et al., 2011).

Similarly to Strategy I species, a bHLH transcription factor, OsIRO2, with homology to AtbHLH38-39, activates the expression of most of the genes related to PS production, secretion and uptake (*NAS1, NAS2, NAAT, DMAS, TOM1, YSL15*; Figure 2), and genes involved in the methionine cycle (Kobayashi and Nishizawa, 2012). Other Fe deficiency-induced bHLH genes in rice are *OsIRO3*, with similarity to *AtPYE*, and *OsbHLH133* (Zheng et al., 2010; Kobayashi and Nishizawa, 2012; Wang et al., 2013c).

The expression of the master regulator *OsIRO2* is strongly induced under Fe deficiency (Ogo et al., 2006) and is positively regulated by the IDEF1 transcription factor (Figure 2; Kobayashi et al., 2009). IDEF1 is an ABI3/VP1 transcription factor (Kobayashi et al., 2007) that can bind Fe^2+^ and Zn^2+^ (Kobayashi and Nishizawa, 2015), and is especially important for the early response to Fe deficiency (Kobayashi et al., 2009). IDEF2 is a NAC transcription factor (Ogo et al., 2008) that regulates OsYSL2 and other Fe deficiency-inducible genes, which may be involved in Fe translocation (Kobayashi et al., 2014). Both IDEF1 and IDEF2 are constitutively expressed in vegetative and reproductive tissues without induction by Fe deficiency (Kobayashi and Nishizawa, 2012).

The role of ethylene and other hormones in the regulation of Fe deficiency responses has been less studied in Strategy II species. In rice, as occurred in Strategy I species (see previous Section), several hormones (ethylene, auxin, jasmonic acid, ABA) have been implicated in the regulation of its Fe deficiency responses (reviewed by Kobayashi et al., 2014). Very recently, Shen et al. (2015) have shown that OsARF16, a transcription factor regulating auxin redistribution, is required for Fe deficiency response in rice.

In relation to ethylene, the few existing data do not support an important role for this hormone in the regulation of responses to Fe deficiency in Strategy II species. Firstly, roots from several Fe-deficient Strategy II species did not produce more ethylene than the Fe-sufficient ones, as observed in Strategy I species (Romera et al., 1999; Wu et al., 2011; see Section Ethylene Synthesis and Signaling in Rice and Strategy II Species). Secondly, the addition of ACC to barley plants did not increase either the production of PS (Welch et al., 1997) or the expression of Fe-related genes (Wu et al., 2011). In the case of rice, Zheng et al. (2009) and Wu et al. (2011) have found that ethylene production, as well as the expression of some ethylene synthesis genes, increase under Fe deficiency (see Section Ethylene Synthesis and Signaling in Rice and Strategy II Species). Wu et al. (2011) have also shown that ethylene is implicated in the activation of some Fe-related genes, such as *OsIDEF1, OsIRO2, OsITR1, OsYSL15, OsNAS1*, and *OsNAS2* (Figures 2, 3). Some of these genes are generally associated to the Strategy I system, like *OsITR1*, while other ones are specifically associated to the Strategy II system, like *OsIDEF1, OsIRO2*, and *OsYSL15* (Kobayashi and Nishizawa, 2012; Kobayashi et al., 2014). Moreover, as in Strategy I species, the addition of ACC to Fe-sufficient plants upregulates the expression of the above genes to a lesser degree than when applied to Fe-deficient plants (Wu et al., 2011; see Section Are Physiological and Morphological Responses Regulated Similarly by Ethylene?).

Ethylene, NA and PS are synthesized from L-methionine, and some genes, e.g., *MTK, SAMS* and *NAS*, are induced by Fe deficiency in both Strategy I and Strategy II species (Figure 3; see Section Ethylene Synthesis and Signaling under Fe Deficiency). This implies that both strategies could share the first steps in the regulation of their responses to Fe deficiency while differ in the last steps. Probably, both kind of plants have diverged along the evolution in such a way that Strategy I species have devoted SAM to NA and ET synthesis while Strategy II species have devoted SAM to NA and PS synthesis (Figure 3).

## Cross talks between Fe deficiency and other nutrient deficiencies. ¿is ethylene a common regulatory signal for different nutrient deficiencies?

Although the responses to Fe deficiency are specifically induced under the deficiency of this metal, it is relatively frequent to find induction of some Fe responses under other nutrient deficiencies. On a reciprocal basis, frequently the induction of responses to other nutrient deficiencies occur under Fe deficiency (Table 1). These cross talks among nutrient deficiencies could be related to the common participation of similar regulatory signals, like ethylene, auxin and NO, in the induction of their responses (reviewed in García et al., 2015). In supporting this view, ethylene has also been implicated in the activation of responses to P deficiency (Lei et al., 2011; Nagarajan and Smith, 2012; Wang et al., 2014b), to K deficiency (Jung et al., 2009), to S deficiency (Moniuszko et al., 2013), and to other deficiencies (García et al., 2015 and references therein; See other reviews in this Issue).

**Table 1:** **Cross talk between Fe deficiency and other nutrient deficiencies**.

**Name**	**Nutrient deficiency**	**References**
**Fe RESPONSES OR Fe-RELATED GENES INDUCED UNDER OTHER NUTRIENT DEFICIENCIES (Str. I)**		
Ferric Reductase Activity	-P, -Cu	Romera et al., 2003; Bernal et al., 2012; Wang et al., 2014b
*FRO* (Ferric reductase)	-P, -S, -Cu	Abel, 2011; Bernal et al., 2012; Muneer et al., 2014; Wang et al., 2014b
*IRT* (Fe^2+^ transporter)	-P, -S, -K, -Cu	Wang et al., 2002; Abel, 2011; Bernal et al., 2012; Muneer et al., 2014; Wang et al., 2014b
Acidification	-P	Wang et al., 2014b
Phenolics	-P	Wang et al., 2014b
Organic acids	-P	Wang et al., 2014b
Root hairs	-P, -K	Jung et al., 2009; Wang et al., 2014b
**NUTRIENT-RELATED GENES INDUCED UNDER Fe DEFICIENCY (Str. I)**		
*SlPT1* (P transporter)	-Fe	Wang et al., 2002
*SlKCl* (K channel)	-Fe	Wang et al., 2002
*AtSULTR1; 1* (Sulfate transporter)	-Fe	García et al., 2010
*SlST1.1* (Sulfate transporter)	-Fe	Paolacci et al., 2014
*SlST1.2* (Sulfate transporter)	-Fe	Paolacci et al., 2014
*SlST2.1* (Sulfate transporter)	-Fe	Paolacci et al., 2014
*AtCOPT2* (Cu transporter)	-Fe	Colangelo and Guerinot, 2004; García et al., 2010; Perea-García et al., 2013
**NUTRIENT-RELATED GENES INDUCED UNDER Fe DEFICIENCY (Str. II)**		
S assimilatory pathway genes	-Fe	Ciaffi et al., 2013

*Str. I, Strategy I; Str. II, Strategy II*.

Several Fe deficiency responses are up-regulated by P, S, K, or Cu deficiency in Strategy I species (Table 1; Wang et al., 2002, 2014b; Romera et al., 2003; Abel, 2011; Bernal et al., 2012). Under P deficiency, plants can induce changes like proliferation of root hairs, cluster roots, increased exudation of phenolics, citrate and protons, and an increased ferric reductase activity, strongly resembling the Fe deficiency response of Strategy I species (Wang et al., 2014b). Some Fe acquisition genes, such as *IRT1*-like, *FRO2*-like and *FIT*-like genes (Figure 1), were upregulated by P deficiency in Arabidopsis, tomato, and lupin plants (Wang et al., 2002; Abel, 2011 and references therein; Wang et al., 2014b). The up-regulation of Fe responses by P deficiency is further supported by the higher Fe accumulation in P-deficient plants (Ward et al., 2008). In oilseed rape, the Fe acquisition genes *BnIRT1* and *BnFRO1* were up-regulated by S deficiency during the earlier stages of the deficiency (Muneer et al., 2014). Similarly, *SlIRT1* was up-regulated by K deficiency in tomato plants (Wang et al., 2002). K deficiency also induces proliferation of subapical root hairs where ethylene has been involved (Jung et al., 2009). Ferric reductase activity was induced by Cu deficiency in soybean and other Strategy I species (Romera et al., 2003 and references therein). This response, as well as *AtIRT1* and *AtFRO2* expression, was also induced in Cu-deficient Arabidopsis plants in a SPL7 (SQUAMOSA PROMOTER BINDING PROTEIN-LIKE7) dependent manner, being induced higher in Cu-deficient *spl7* mutant plants than in the wild type (Bernal et al., 2012). The data suggest that Cu deficiency leads to a lower translocation of Fe from roots to shoots in the *spl7* mutant. This results in greater Fe deficiency in the *spl7* shoots, which in turn triggers Fe deficiency responses (Bernal et al., 2012). As seen above, different nutrient deficiencies can induce Fe responses. On the other hand, Fe deficiency can up-regulate responses to other nutrient deficiencies (Table 1; Wang et al., 2002; García et al., 2010; Perea-García et al., 2013). As examples, the tomato P transporter, *SlPT1*, and the K channel, *SlKC1*, were up-regulated in roots of Fe-deficient tomato plants (Wang et al., 2002). Similarly, sulfate transporters, like *AtSULTR1;1* in Arabidopsis (García et al., 2010), and *SlST1.1, SlST1.2*, and *SlST2.1* in tomato (Paolacci et al., 2014), were induced under Fe deficiency. In the same way, the Cu transporter, *AtCOPT2*, was induced under Fe deficiency in Arabidopsis (Colangelo and Guerinot, 2004; García et al., 2010; Perea-García et al., 2013). These cross talks among deficiencies also occur in Strategy II species: in wheat, several genes of the S assimilatory pathway induced by S deficiency were also significantly up-regulated by Fe deficiency (Ciaffi et al., 2013).

Besides these mutual and positive influences among nutrient deficiencies, some elements, either under deficiency or excess, can negatively affect the responses to Fe deficiency. As examples, S deficiency, depending on its severity and extent, can limit the development of Fe responses in Strategy I species, like tomato (Zuchi et al., 2009) and oilseed rape (Muneer et al., 2014). Similarly, S deficiency can also limit Fe responses in Strategy II species, like the release of PS (Astolfi et al., 2006). Interestingly, S, through methionine and its derivatives, participates in the synthesis of NA, PS, and ethylene (Figure 3; Sauter et al., 2013). Co excess can cause Fe deficiency by inhibiting Fe responses, which is logical since Co is a potent ethylene inhibitor (Romera and Alcántara, 1994, 2004 and references therein). Other heavy metals, like Ni, Cu and Cd, when applied at concentrations between about 5–20 μM in nutrient solution, can block the induction of some Fe physiological responses (Alcántara et al., 1994). Nonetheless, the relationship of these heavy metals with ethylene needs further research.

Besides the above interactions between Fe and other nutrients, there are intriguing cross talks between Fe responses and some defense responses, like the ISR (Induced Systemic Resistance). This latter cross talk suggests that responses to biotic and abiotic stresses can share some common regulatory components. ISR is a mechanism by which selected plant growth-promoting bacteria and fungi in the rhizosphere prime the whole plant body for defense against a broad range of pathogens and insect herbivores (Pieterse et al., 2014). Some Fe-related genes, like *AtMYB72, AtBGLU42, AtABCG37, AtFRO2, AtIRT1* and others (Figure 1), are upregulated in Arabidopsis roots colonized by ISR-inducing *Pseudomonas* strains (Pieterse et al., 2014; Zamioudis et al., 2014). Using the Arabidopsis ethylene mutant *eir1*, which is insensitive to ethylene in roots, it was shown that ethylene is required for the expression of ISR (Pieterse et al., 2014). The common implication of ethylene in the regulation of Fe deficiency responses and ISR could partially explain the cross talk between both processes.

## Conclusions

The participation of ethylene in the activation of most of the morphological and physiological responses to Fe deficiency in Strategy I species implies that it acts as a general coordinator of their control. Nonetheless, morphological and physiological responses seem to be regulated by ethylene through different signaling pathways. Additionally, several results suggest that ethylene acts in conjunction with other positive signals, like auxin and NO, and with negative signals, e.g., probable Fe-related repressive signals moving through the phloem. Ethylene has also been implicated in the regulation of some Fe responses in rice, that possesses combined characteristics of both Strategy I and Strategy II species. In Strategy II species, the few existing data do not support an important role for ethylene in the regulation of their Fe deficiency responses. The common involvement of ethylene in the regulation of responses to other nutrient deficiencies and in the regulation of the ISR, could partially explain the cross talk between Fe deficiency responses and responses to other deficiencies, and Fe deficiency responses and the ISR.

## Conflict of interest statement

The authors declare that the research was conducted in the absence of any commercial or financial relationships that could be construed as a potential conflict of interest.
