# Supplementary material for: Ethylene Participates in the Regulation of Fe Deficiency Responses in Strategy I Plants and in Rice
Source: Front Plant Sci. 2015 Nov 27;6:1056. doi: 10.3389/fpls.2015.01056 (PMC4661236; doi:10.3389/fpls.2015.01056)
Supplement: Supplementary file 1 [file DataSheet1.PDF]

subapical root hairs by Fe-deficient plants. Additionally, these results show that different ethylene receptors could be involved in different processes.

The use of ethylene mutants to demonstrate a role for this hormone in the regulation of other Fe-deficiency stress

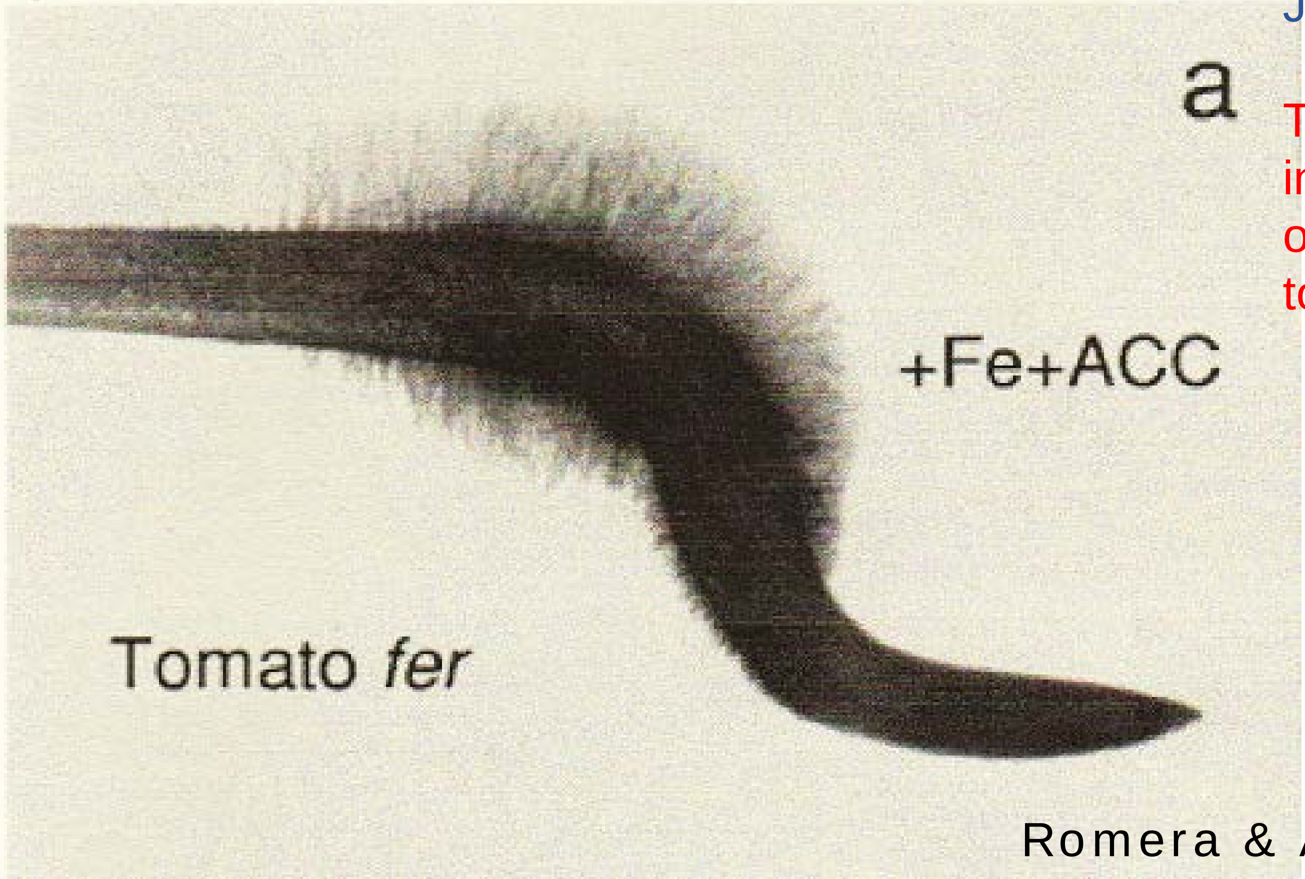

Justification to Fig. 5

The addition of ACC induces the formation of root hairs in the tomato *fer* mutant

Romera & Alcántara, 2004

Justification to Fig. 6

The addition of an ethylene inhibitor (Co) diminishes Fe concentration in shoots and, consequently, toxicity symptoms (see next page)

E107 = *brz*

Table 1. Effects of Co^{2+} on *E107* pea root Fe(III)-chelate reductase activity, shoot iron concentration, and shoot and root fresh weights (FW) of 19 day old, iron-sufficient seedlings

Treatment	Reductase activity (μmol Fe^{2+} g RFW^{-1} h^{-1})	Shoot [Fe] (μg g DW^{-1})	Shoot FW (g)	Root FW (g)
+Fe	1.45 (±0.19)	849 (±82)	2.6 (±1.1)	2.3 (±1.3)
+Fe, +Co	0.44 (±0.20)	193 (±23)	4.5 (±1.1)	5.1 (±0.5)

E107 seedlings were grown in nutrient solution supplied with 20 μM Fe(III) EDDHA from day 11. On day 12, 3 μM $CoCl_2$ was supplied to the cobalt treatment; mean ± SEM ($n = 6$).

responsible for Fe(III)-chelate reductase expression directly. However, ethylene may influence the expression of other genes prior to the induction of Fe(III)-chelate reductase synthesis, such as the genes involved in root morphological changes (e.g. root hair development). We have observed that

ACC promotes the development of ro[illegible] swollen tips'), which is a prerequis[illegible] Fe(III)-chelate reductase activity in roo[illegible] under iron-deficient conditions. This ob[illegible] the latter possibility.

Romera et al., 1996

Effects of cobalt and AOA on Fe(III)-chelate reductase activity in iron-sufficient seedlings

These experiments were conducted only with *E107* and *chloronerva* mutants because their normal parental genotypes suppress Fe(III)-chelate reducing capacity when supplied adequate iron (Scholz *et al.* 1988, Grusak *et al.* 1990, Welch & La Rue 1990). The addition of Co^{2+} ions (3 μM) to the nutrient solution of iron-sufficient *E107* pea seedlings inhibited the development of root Fe(III)-chelate reducing capacity (Table 1). On day 18, the oldest leaves of the untreated iron-sufficient *E107* pea plants showed severe iron toxicity symptoms (i.e. leaf bronzing and inter-venial necrosis) while toxicity symptoms were absent in older leaves of iron-sufficient plants treated with cobalt. Toxicity symptoms were correlated with the iron concentration in shoots, with iron concentrations substantially lower in shoots of cobalt-treated plants (Table 1). The cobalt-treated, iron-sufficient seedlings grew normally and had higher root and shoot fresh weight than those plants not treated with cobalt.

Justification to Fig. 6

Romera et al., 1996

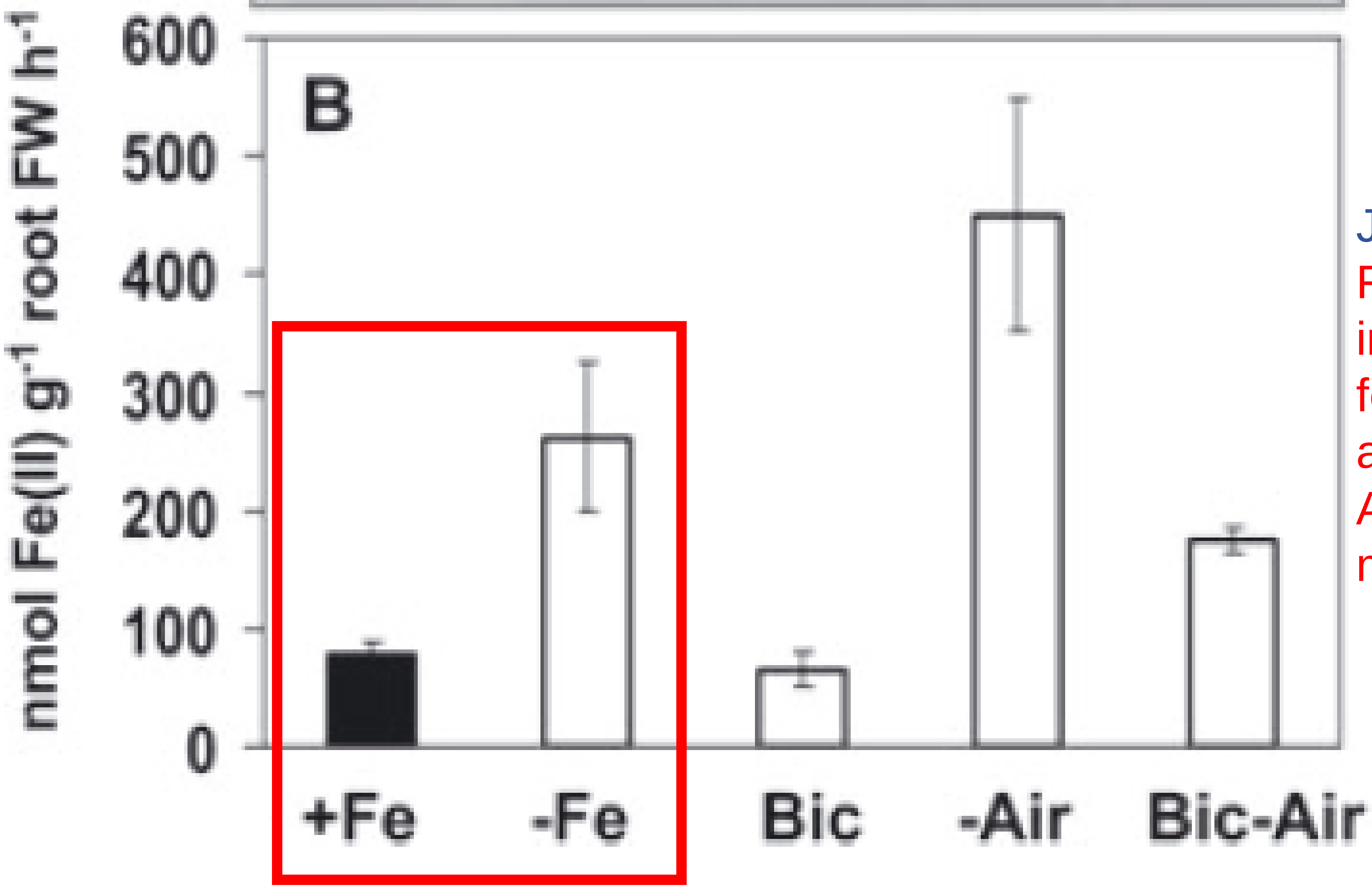

Justification to Fig. 8
Fe deficiency induces enhanced ferric reductase activity in the Arabidopsis *ctr1* mutant

Fig. 4. Effects of bicarbonate and hypoxia on the expression of the Fe acquisition genes *AtFRO2*, *AtIRT1*, *AtFIT*, *AtbHLH038* and *AtbHLH039* (A) and on the ferric reductase activity (B) of Fe-deficient *Arabidopsis ctr1* plants. Treatments and determinations as in Fig. 2.

García et al, 2014

Physiol. Plant. 150, 2014
